# Improvement of innate immune responses and defense activity in tiger shrimp (*Penaeus monodon* Fab.) by intramuscular administration of the outer membrane protein *Vibrio alginolyticus*

**DOI:** 10.1186/2193-1801-2-432

**Published:** 2013-09-03

**Authors:** Maftuch, E Prasetio, A Sudianto, M Rozik, R Nurdiyani, E Sanusi, H Nursyam, F Fariedah, Marsoedi, Murachman

**Affiliations:** Fisheries and Marine Science Faculty, Brawijaya University, Malang, East Java Indonesia; Fisheries Science Faculty, Palangkaraya University, Kalimantan, Indonesia

**Keywords:** Outer membrane protein, Immunomodulation, Tiger shrimp, *Vibrio harveyi*

## Abstract

The *Outer Membrane Protein* (OMP) of *Vibrio alginolyticus* cell wall was administered intramuscularly (IM) to the tiger shrimp (*Penaeus monodon* Fab.) at 10, 20, 30 μg/kg bw. After 14 days infection, the tiger shrimps were challenged with 10^7^ bacterial density of *Vibrio harveyi* for 24 hours. The total haemocyte count (THC), differential haemocyte count (DHC) and amount of total protein plasma (TPP), superoxide dismutase and protease enzyme activity were monitored. The results showed that intramuscular administration of OMP enhanced an immunomodulatory effect and protection against *V. harveyi*. The beneficial effect of OMP on the tiger shrimp is dose-dependent and OMP-20 μg/kg bw is an optimal dose after two times of boosters for 14 days against *V. harveyi* infection.

## Introduction

Tiger shrimp (*Penaeus monodon* Fabricus) are one of the most important aquaculture products in several countries, especially in South-East Asia. But in Indonesia, tiger shrimp have almost entirely disappeared because of diseases. Disease problems in aquaculture are caused by environmental problems such as poor water conditions, leading to stress and disease outbreaks (Sadovy, 2000). The big disease outbreaks in tiger shrimp aquaculture are caused by viruses and bacteria e.g., *Vibrio* spp. Therefore, control of diseases is an important task for the further development of tiger shrimp culture.

There are many techniques widely used to control the diseases, such as using antibiotics and chemotherapy. However, experience demonstrates that there are several problems associated with the use of antibiotics and chemotherapy in clinical treatment of shrimp diseases. Such as, potential environmental hazards, the spread of antibiotic-resistant bacteria and associated stress (Jin et al. 2008). Thus, researchers have focused on finding alternative methods of disease control such as the application of vaccines and immunostimulants of microbial origin (Sakai, 1999). The OMP is an effective microbial origin immunostimulant and has been successfully used to strengthen the non-specific defense systems of shrimp and also non-specific and specific defense systems of fish (Maftuch, 2006). The OMP was isolated from the *Vibrio alginolyticus* cell wall.

Many reports showed that OMP contain LPS, beta-glucan and proteins that can enhance macrophage phagocytic activity, which is part of the non-specific defense system (Jin et al. 2008). However, there are a few reports about the application of OMP in Shrimp. The purpose of the study was to explore the effect of OMP on the immune response in tiger shrimp, as well as on resistance against *Vibrio harveyi.*

## Materials and methods

### Preparations of outer membrane (OMP) from *V. alginolyticus*

*V. alginolyticus* strains were isolated from pathogenic shrimp (*Penaeus monodon* Fab.) that cultured in marine ponds at the Research Centre of Brackish Water, East Java Province, Indonesia. The isolate was cultured on thiosulfate-citrate-bile salt-sucrose (TCBS) agar plates. Therefore the *V. alginolyticus* was grown in 1000 ml Brain Heart Infusion Broth (BHIB) at 30°C for 24 hours, and then were separated into 100 bottles containing Thiaproline Carbonate Glutamate (TCG) medium at 30°C, for 48 hours to enrich growth pili of *V. alginolyticus*. The medium contains 0.02% Thioproline, 0.3% NaHCO3, 0.1% monosodium l-glutamate, 1% bactotryptone, 0.2% yeast extract, 0.5% NaCl, 2% bacto agar and 1 mM Beta-amino ethyl ether N,N,N,“n”-tetra acetic acid (EGTA).

OMP was isolated from both pili (growth medium) and cell membrane (cell pellet), following (Garavito and Rosenbusch 1986). The pili were isolated using micro centrifugation (3,500 g, 15 min, 4°C) six times. The Pili’s content was put in the solution. The cell pellet (1 gram) was resuspended in 15 ml of lysis buffer. After incubation for 1 min with gentle shaking and centrifugation at 12,000 g and 4°C for 30 min, the solution containing the membrane fraction was dialyzed for 24 hours by dH_2_O and following with Phosphate Buffer Saline (PBS) pH 7.4 for 24 hours. The determination of protein concentration was estimated using the BCA assay kit (Pierce, Rockford, IL, USA) according to the manufacturer’s instructions. A protein sample (12.5 μl) was mixed with 100 μl of the BCA working reagent. After the reaction mixture was incubated at 37°C for 30 min, absorbance at 540 nm was measured with a microplate reader spectrophotometer (Labsystem, Finland). BSA at various concentrations, ranging from 0.025 to 1.0 mg/ml, was used to construct a standard calibration curve and to determine protein concentrations of the samples.

### Preparation of *V. harveyi*

*V. harveyi* strain was isolated from pathogenic shrimp (*P. monodon* Fab.) from marine ponds at the Research Centre of Brackish Water, East Java Province, Indonesia. The isolate was cultured on thiosulfate-citrate-bile salt-sucrose (TCBS) agar plates, and then grown in 1000 ml of Brain Heart Infusion Broth (BHIB) at 30°C for 24 hours. The harvested bacteria were washed 3 times in 1.5% physiological saline by centrifugation at 1,000 g for 10 min. The concentration of bacterial suspension was determined by spectrophotometer and diluted to 10^9^ cell/ml in 1.5% sterile physiological saline for preparation.

### Shrimp immunization and bacterial challenge

A total two hundred and fourty tiger srimps were divided into four groups, each group comprised 60 tiger shrimps were immunized by OMP 10, 20, 30 μg/kg bw, and control (without immunized). The Immunizations were done by injecting OMP directly in the first swimming leg of the abdomen using a 1 mL syringe equipped with a 22 gauge needle. Second injection or boostering was done after 7 days first immunization. After 14 days immunization, the srimps were chalanged with *V. harveyi* for 24 hours. Sixty tiger shrimps for each treatment were cultured at 10 liters of water diluted with *V. harveyi* 10^7^ cell/ml in culture media for 24 hours. Another 60 tiger shrimps without immunized with OMP were cultured in standard media as control. The mortality was monitored for 1 day after infection (Jin et al. 2008). Haemolymph was collected after 24 hours infection, from abdomen sides in the second legs of shrimp by using a 1 ml syringe equipped with a 22 gauge needle. The Haemolymph was stored at room temperature and ready to be used for sample of each parameters assay (Johansson et al. 2000).

### Total haemocyte count (THC)

The THC was measured according to modified method from (Wootton et al. 2003). A hundred μl of haemolymph was transferred into a tube which contained 900 μl of Natt-Herricks’s stain solution. A drop of haemolymph collected from each individual was introduced into an improved Neubauer hemocytometer and the number of haemocytes was determined microscopically. THC was calculated using the following formulation (Wootton et al.^2003^):

### Differential haemocyte counts (DHCs)

The DHCs was performed according to (Wootton et al.^2003^). The haemolymph of shrimp was collected (100 μl) and diluted with 100 μl of TBS buffer, then a total volume of 0.2 ml (200 μl). The cells solution was mixed well and 200 μl was spread on a glass slide and placed at room temperature for 60 minutes. Then, the cells were fixed using Baker’s formol calcium (+ 2% NaCl). After 30 minutes, the glass slide was rinsed with absolute methanol for 5 minutes. The cells were stained with H&E staining cells (pH 6.6) for 5 minutes. Afterward, the slide was washed with dH_2_O. The cell was observed under light microscope using 100× objective lenses. A minimum of 100 haemocytes per slide were examined to determine the DHC.

### The amount of protein plasma assay

The *in vitro* amount of TPP of the haemocytes of *P. monodon* was assessed by following the modified procedure of (Van de Braak, 2002). A hundred microliters of haemolymph were centrifuged at 1500 rpm, at 4°C, for 10 minutes. Then 0.05 ml supernatant was mixed by adding of 1 mg/ml Bovine Serum Albumin (BSA), aquades 4.75 ml, 0.5 ml Alk.CuSO_4_ and 0.05 ml Lowry Reagent medias, and by following the modification procedure of (Lowry et al. 1951). The values were expressed as O.D. at 660 nm/15 min.

### Superoxide dismutase (SOD) activity assay

Superoxide Dismutase activity Assay, considered as its ability to inhibit superoxide radical, was done by previously method (Chandra et al. 2006). One unit of SOD activity was defined as the amount required to inhibit the rate of xanthine reduction by 50% (Jin et al. 2008). Specific activity was expressed as SOD unit/mg protein.

### Protease enzyme activity assay

Protease enzyme activity assay was assessed by tyrosine reduction following the modification procedure of (Celis-Guerrero et al. 2004). One unit protease activity was counted by converting the value of absorbance to tyrosine concentration (Celis-Guerrero et al. 2004).

### Statistical analysis

All the immune parameters analysis was performed with the SPSS 15 statistical software. One-way analysis of variance (One-way ANOVA) was applied to analyze the differences among treatments and control. Then, a post hoc test (Duncan) was conducted to display the mean for groups in the homogenous subset.

## Results and discussion

**Figure 1:**
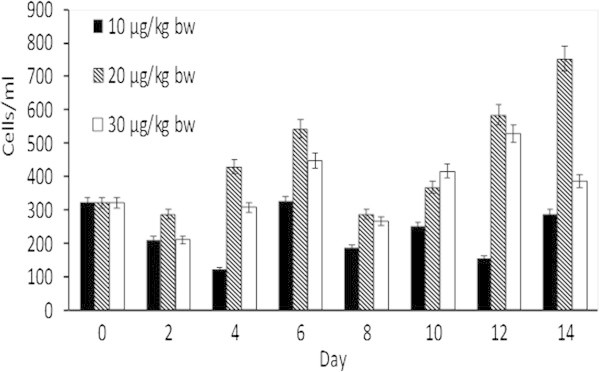
**Effects of different dosages of intramuscular administration of OMP on total haemocyte count (THC) of tiger shrimp (×10**^**4**^**sel/mm**^**3**^**) for fourteen days.** Data are represented as mean ± S.D. (n = 10).

The Tiger shrimp was administered by OMP intramuscularly (IM) at 10, 20, 30 μg/kg bw. The THC was collected every two days from day 2 until day 14 after imunization. The data showed that after day 2 administered with OMP decreased THCs compared to control (day 0) and increased again at day 4 and day 6. Futhermore THCs decreased again in day 8, after second imunization, and then increased at day 10, day 12 and day 14. This result indicated that administration OMP 20 μg/kg bw stimulated the highest THC in tiger shrimps compare to other doses (Figure 1). This phenomenone was similar to Johansson statement, stated that ß-glucan treatment as immunostimulant could decrease haemocyte at the moment and then increased dramatically (Johansson et al. 2000). (Yeh et al. 2005) reported that administration of *Sargassum duplicatum* extract could increase haemocyte of *Litopenaeus vannamei*. Haemocyte is part of cells in haemolymph, it has function to protect infectious disease as an immune response of the invertebrate organism (Van de Braak, 2002).

### Total haemocyte count

**Figure 2:**
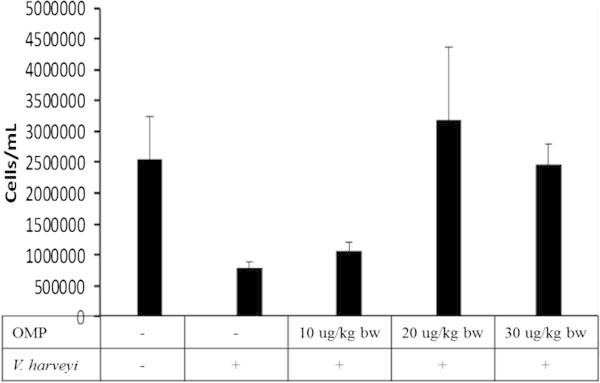
**Effects of different dosages of intramuscular administration of OMP and*V. harveyi*infections on total haemocyte count (THC).** Data are represented as mean ± S.D. (n = 10). It indicates significant differences (P < 0.05) among five treatments determined by one-way ANOVA.

The total haemocyte count (THC) of tiger shrimp was significantly different with intramuscular administration of OMP and *V. harveyi* infections (P < 0.01). Greatly improved THC was observed in the treatment of OMP 20 μg with THC value of 317.5 × 10^4^ cells/ml (Figure 2).

The haemocyte circulation system may cause modulation of THC. Indeed, invertebrates (including shrimp) blood circulation system is open while haemocytes are distributed in both the vascular system and tissues. Consequently, an increase of THC values was assumed from either proliferation or movement of cells from tissues into haemolymphs. The concentration of cells varied as function of the development stage of an intermoult cycle of *P. japonicus* post larvae (Tsing et al. 1989). A decrease of THC may be due to cell lysis or increased movement of cells from haemolymph to tissues (Pipe and Cole, 1995). The decreasing of THC is also presumably due to the altered physiological conditions of the shrimp, as enhanced susceptibility to bacterial infection (Ford et al. 1993).

### Differential haemocyte count

**Figure 3:**
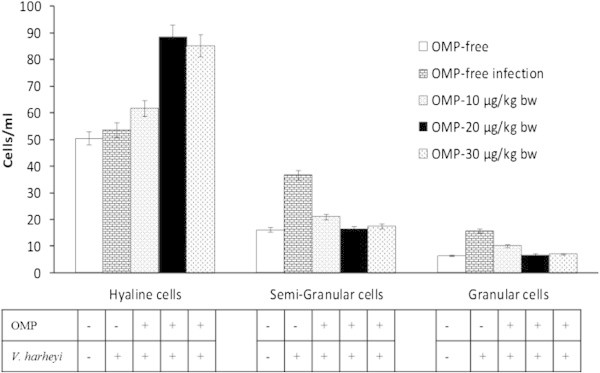
**Effects of different dosages of intramuscular administration of OMP and*V. harveyi*infections on differential haemocyte count (DHC).** Data are represented as mean ± S.D. (n = 10). It indicates significant differences (P < 0.05) among five treatments determined by one-way ANOVA.

The DHC (hyaline, semigranular, granular) of tiger shrimp were given intramuscular administration of OMP and *V. harveyi* infections, where hyaline, semigranular, and granular were significantly different among five treatments determined by one-way ANOVA (Figure 3). Phagocytosis is believed to be one of the major cellular defence mechanisms in crustaceans. The semigranular haemocytes are the primary cells involved in the phagocytosis of foreign particles in shrimp (Bachère et al. 1995). The similar morphological haemocytes were also reported as haemoblasts in *Tapes philippinarum* (Matozzo et al. 2008) and *Saccostrea glomerata* (Aladaileh et al. 2007b). This cells were identified with different functions which do not play a role in defensive responses like phagocytosis or encapsulation, and they lack the common intracellular enzyme systems associated with host defense, unlike hyaline and granular (Aladaileh et al. 2007b).

Granular haemocytes are also capable of phagocytosis of foreign material but with less frequency than the smaller ones (Hose and Martin, 1989). Granular cells have been proven to play a significant role in the shrimp defence system because of their antibacterial activity (Chisholm and Smith, 1995). Granular played a role in phagocytosis, which may be correlated by their capacity for intracellular killing. Moreover, several studies showed that granular contain high levels of acid phosphatase and phenol oxidase enzymatic activities, as well as the ability to form superoxides and peroxides (Xue et al. 2000). The other haemocytes are the hyaline cells. They are also considered as phagocytes and superoxide anions production (Söderhäll and Cerenius, 1992).

The results of the study showed that treatment significantly increased the percentages of hyaline and decreased the percentages of semigranular and granular cells in the haemolymph. It was presumed that the young are generally known as the hyaline cells and that those cells gave rise to two haemocytic developmental series, i.e. the large- and small-granular cell line. Granular cells of the large-granular cell line mature and accumulate in the connective tissue, and many cells of the small-granular cell line were located in the lymphoid organ (Van de braak, 2002). Decreases in the percentages of semigranular and granular cells were compensated for by a proportional increase in the percentages of hyaline population. In the present study, haemocytes may display recruitment of immature cells from haematopoietic tissues (Hine, 1999). (Aladaileh et al. 2007a) predicted that the increased percentages of particular haemocyte types were due to induced cellular proliferation, recruitment of cells from non-circulating compartments of the haemolymph, or rapid cellular differentiation in response to antigenic challenge.

### Total plasma protein

**Figure 4:**
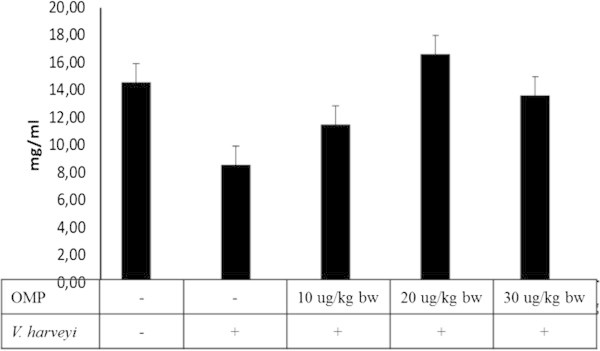
**The Effects of different dosages of intramuscular administration of OMP and*V. harveyi*infections on total plasma protein (TPP).** Data are represented as mean ± S.D. (n = 10). Different letters indicate significant differences (P < 0.05) among five treatments determined by one-way ANOVA.

The total plasma protein (TPP) or haemolymph protein activity of black tiger shrimp was significantly different with intramuscular administration of OMP and *V. harveyi* infections (P < 0.01) (Figure 4). A Great improved TPP was observed in treatment of OMP 20 μg with TPP value of 16.56 mg/ml (Figure 4).

The TPP has been observed in previous studies on shrimp. The changing of TPP composition in the blood was affected with animal size, sex, nutritional state, environmental factors (such as temperature, salinity) and the moult cycle but this was inversely related to haemolymph volume (Chen and Cheng, 1993) and a decrease of TPP during infection as well as during repeated haemolymph sampling (Ford et al. 1993). In this study, TPP increased with treatment of OMP-10 and 20 μg/kg bw but decreased during infection of bacteria and treatment OMP-30 μg/kg bw. Those were similar to (Chen et al. 1994), the TPP increased with treatment of 10 and 20 ppt and decreased with treatment of 30 ppt.

### Superoxide dismutase and protease enzyme activity

**Figure 5:**
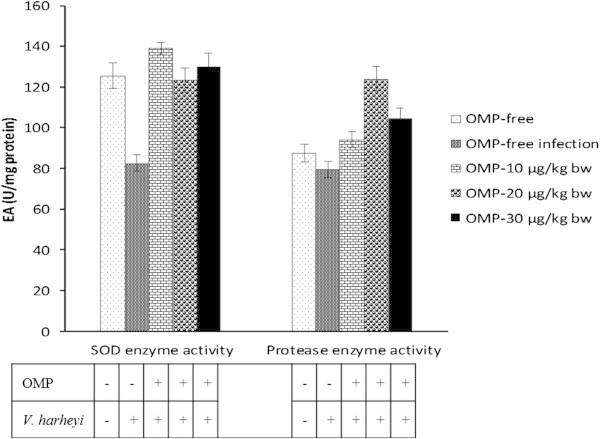
**Effects of different dosages of intramuscular administration of OMP and*V. harveyi*infections on superoxide dismutase (SOD) and protease activity.** Data are represented as mean ± S.D. (n = 10). Different letters indicate significant differences (P < 0.05) among five treatments determined by one-way ANOVA. EA denote enzyme activity (U/mg protein).

The humoral parameters are SOD and protease activities of tiger shrimp. The SOD and protease activities were significantly different with intramuscular administration of OMP and *V. harveyi* infections (P < 0.05) (Figure 5). Greatly improved SOD activity was observed in the treatment of OMP 10 μg with SOD value of 139,09 unit /mg protein and protease activity of OMP 20 μg with protease activity value of 130,17 unit/mg protein (Figure 5).

Concern to haemocytes function, superoxide anion produces toxic oxygen metabolites. Production of toxic oxygen metabolites, such as protease and *reactive oxygen species* (ROS) activation anion superoxide/O_2_^-^, hydrogen peroxide/H_2_O_2_, ion hydroxide/OH^-^ and oxygen/O_2_ (Campa-Cordova et al. 2002) is believed to be mediated through phagocytosis, encapsulation, aggregate nodulation, melanocytes and cytotoxicity which destroy invasive pathogens (Rodriguez and Moullac, 2000), thus providing an explanation for the combined suppression of phagocytosis and superoxide anion (Anderson et al. 1992).

The levels of protease activity were enhanced, caused by intramuscular administration of OMP and *V. harveyi* infections in tiger shrimp. The protease enzyme has a role to play as a lysosomal enzyme and prophenoloxidase (proPO) activator to become a phenol oxidase (PO) enzyme (Van de Braak, 2002). During that process, it was followed by phenol oxidation to become quinone that is antibacterial molecules (Smith et al. 2003). (Yeh et al. 2005) said that immunostimulants could enhance Phenol oxidase enzyme activity. Similar study in *Biomphalaria glabrata* reported that serine protease enzyme activity enhancement was followed by phenol oxidase enzyme activity enhancement (Bahgat et al. 2002).

On the other hand, an alteration in the levels of superoxide anion caused by contaminant exposure has been well studied in invertebrates (Wootton et al. 2003). A study on bivalves reported that bivalves’ superoxide generation is generally enhanced with low concentrations of contaminant exposure, but inhibited at higher concentrations (Dyrynda et al. 2000). In the present study, intramuscular administration of OMP and *V. harveyi* infections presented significant differences from control to produce protease and superoxide anion.

### Mortality

**Figure 6:**
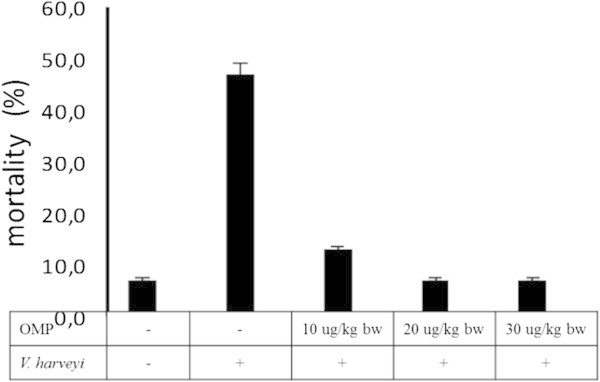
**Cumulative mortality (%) of tiger shrimp after two times of boosters of OMP for 14 days against*V. harveyi*infection for 24 hours.**

Initial mortality tiger shrimp occurred on the next day after challenge. The highest cumulative mortality (46%) was observed in tiger shrimp with the control infection (OMP-free infection) (Figure 6). While shrimp with various OMP injection had reduced mortality until of 6-13%. Similar results had been reported in previous studies on shrimp when using immunostimulant (beta glucan) (Huang et al. 2006). In this study, treatment of OMP-20 μg/kg bw by intramuscular injection showed the most efficient and desirable influence on resistance of tiger shrimp to *V. harveyi.* However, treatments of OMP-10 μg/kg bw and OMP-30 μg/kg bw reduced of mortality of tiger shrimp but they were not more efficient than treatment of OMP-20 μg/kg bw. It probably concluded that the beneficial effect of OMP of disease resistance of tiger shrimp is dose-dependent and OMP-20 μg/kg bw is an optimal dose after two times of boosters of OMP for 14 days against *V. harveyi* infection for 24 hours.

## Conclusion

The research demonstrated that OMP *V. Alginolyticus* administrated intramuscularly enhance immune responses as well as the resistance to *V. harveyi* in tiger shrimp. In this research, the optimal immune parameters were observed in tiger shrimp induced by OMP-20 μg/kg bw.
